# Locally Recurrent Rectal Cancer in the Molecular Era: A Scoping Review of Biomarkers of Oncologic Outcome following Salvage Surgery

**DOI:** 10.1245/s10434-026-20116-2

**Published:** 2026-07-28

**Authors:** Jennifer K. Vu, Evonne Younan, Sascha Karunaratne, Sarah Sutherland, Paul A. Sutton, Y. Nancy You, Oliver Peacock, Daniel Steffens, Michael J. Solomon, Kilian G. M. Brown

**Affiliations:** 1 Department of Colorectal Surgery, Royal Prince Alfred Hospital and Chris O’Brien Lifehouse, Sydney, Australia; 2 Surgical Outcomes Research Centre (SOuRCe), RPA Institute of Academic Surgery, Sydney, Australia; 3 Faculty of Medicine and Health, The University of Sydney, New South Wales, Australia; 4 Department of Medical Oncology, Chris O’Brien Lifehouse, Sydney, Australia; 5 Colorectal and Peritoneal Oncology Centre, The Christie NHS Foundation Trust, Manchester, UK; 6 Division of Cancer Sciences, The University of Manchester, Manchester, UK; 7 Department of Colon and Rectal Surgery, University of Texas MD Anderson Cancer Center, Houston, TX USA; 8 Division of Colon and Rectal Surgery, Mayo Clinic, Rochester, MN USA; 9 NHMRC Clinical Trials Centre, The University of Sydney, New South Wales, Australia

**Keywords:** Molecular biomarkers, Oncologic outcomes, Surgery, Locally recurrent rectal cancer

## Abstract

**Background:**

Radical salvage surgery offers the only chance of cure for patients with locally recurrent rectal cancer (LRRC), yet up to 50% develop disease recurrence after surgery. Identification of those at highest risk of disease recurrence may improve selection for surgery and intensification of neoadjuvant treatment. This scoping review aimed to identify molecular determinants of oncologic outcomes following surgery for LRRC.

**Materials and Methods:**

This study was conducted in accordance with the Joanna Briggs Institute (JBI) methodology for scoping reviews. MEDLINE, Embase, CENTRAL, and Scopus databases were searched for articles published between 2005 and 2025 that reported associations between blood, tissue, or other molecular factors and postoperative oncologic outcomes (recurrence and survival) in patients undergoing treatment for LRRC.

**Results:**

The search identified 2799 articles, of which 17 were included. Sample size varied from 21 to 213 patients. Locoregional recurrence rates after surgery ranged from 15% to 52% (various timepoints between 3 and 5 years), while 5-year overall survival ranged from 22% to 53%. Elevated carcinoembryonic antigen (CEA) was associated with shorter overall survival in 11 studies, and poorer relapse-free survival in 1 study. Elevated serum carbohydrate antigen 19-9 (CA19-9) was associated with shorter overall survival and disease-specific survival in two studies. No studies evaluating contemporary biomarkers (mutational profile, microsatellite instability, or circulating-tumour DNA) were identified.

**Conclusions:**

Evidence evaluating molecular biomarkers in LRRC is limited and has focused on traditional serum biomarkers such as CEA and CA19-9. Contemporary colorectal cancer biomarkers including mutational profile and ctDNA are yet to be studied in this population.

**Supplementary Information:**

The online version contains supplementary material available at https://doi.org/10.1245/s10434-026-20116-2.

Exenterative salvage surgery offers durable disease control and the only chance of cure for patients with locally recurrent rectal cancer (LRRC) and is therefore considered the standard of care. Although long-term survival is now achievable in up to 55% of selected patients, the risk of subsequent disease recurrence is considerable, reported to be as high as 55% in contemporary series.^1–9^ Early recurrence is particularly devastating, given the magnitude of pelvic exenteration surgery, which is associated with a prolonged recovery and significant functional implications.^1,10–12^ This morbidity is justified by the reasonable chance of long-term disease control or cure (achieved in 45–55% of selected patients in contemporary series), yet predicting early failure, and identifying patients who may undergo potentially futile surgery, is difficult.^3,8,13,14^ Furthermore, whether intensification of multimodal therapy by integration of re-irradiation, induction chemotherapy, and adjuvant chemotherapy or targeted biological therapy may improve oncologic outcomes remains unclear. Improved risk stratification to identify patients at greatest risk of recurrence following salvage surgery may provide an opportunity to improve patient selection and ensure that (neo)adjuvant therapies are directed toward patients most likely to benefit, thereby improving oncologic outcomes.

Despite recent advances in understanding molecular determinants of outcomes in colorectal cancer more generally, and particularly in patients with stage IV disease, there has been comparatively little work done evaluating prognostic and predictive biomarkers in patients with LRRC.^15,16^ Studies in primary and locally advanced rectal cancer have explored potential biomarkers such as KRAS, BRAF, TP53, microsatellite instability, and ctDNA to predict therapy response and prognosis, however such studies have typically excluded patients with LRRC.^17,18^

The objective of this scoping review was to identify all previously evaluated molecular determinants and biomarkers predictive of oncologic outcomes or response to treatment in patients with LRRC. Given the limited and heterogeneous evidence in this area, a scoping review methodology was selected to systematically map the existing literature and identify gaps to inform future research.

## Materials and Methods

This scoping review was conducted in accordance with the Preferred Reporting Items for Systematic Reviews and Meta‐Analyses Extension for Scoping Reviews (PRISMA‐ScR) checklist and the Joanna Briggs Institute (JBI) methodology for scoping reviews.^19,20^

### Protocol and Registration

The review protocol was registered with the Open Sciences Framework on 13 October 2025 (https://osf.io/a36qb/overview).

### Eligibility Criteria

The population/concept/context (PCC) framework was used to develop the search strategy. The population was patients who had undergone surgery for LRRC with curative intent. The concept was molecular factors (e.g., immunophenotypic, genetic, epigenetic, proteomic, metabolomic, transcriptomic, or tumour microenvironment markers) associated with oncologic outcomes (e.g., survival, disease recurrence). The context was curative-intent management in any clinical or research setting, with no geographic restrictions. Articles were included if they contained data relating to associations between blood, tumour tissue, saliva, stool, peritoneal fluid, urine, or other molecular tumour factors and oncologic outcomes.

Databases were interrogated for articles published from 1 January 2005 to 1 October 2025. The search was limited to studies published after 1 January 2005 to ensure results reflect the contemporary understanding of molecular medicine and techniques. No language restrictions were applied, and efforts were made to translate any non-English studies to English. The following study types were included: randomized trials, cohort studies (retrospective and prospective), qualitative studies, abstracts, and published conference proceedings. Case reports were excluded.

### Information Sources

A systematic search was conducted across four databases: MEDLINE, Embase, Cochrane Central Register of Controlled Trials (CENTRAL), and Scopus.

### Search

A three-step strategy was used, beginning with an initial limited search of MEDLINE and Embase via Ovid based on the population/concept/context described above to identify relevant articles. The text words contained in the titles and abstracts of relevant articles, and the index terms used to describe the articles, were used to develop a full search strategy in consultation with a University of Sydney medical librarian for Medline, Embase, CENTRAL, and Scopus. The reference lists of all included studies were screened to identify additional relevant studies, and a gray literature search was conducted using identified sources and those cited in related systematic reviews of a similar topic. If a full text was not found, the authors were to be contacted, however this was not required during this review. The search strategy (included as Supplementary Information), including all identified keywords and index terms, was adapted for each included database and/or information source.

### Selection of Sources of Evidence

Two independent reviewers screened the titles and abstracts against the inclusion criteria. Two reviewers (J.V., E.Y.) independently assessed full texts against the inclusion criteria for eligibility. Reasons for exclusion were recorded. Disagreements were resolved by involving a third reviewer (K.G.M.B.). Two reviewers reviewed the extracted data. Discrepancies were resolved through discussion or consultation with an additional reviewer. Studies that were published in duplicate were only included once, but all versions were to be considered for maximal data extraction.

### Data Charting Process

Citations identified through the search were imported into Covidence systematic review software (Veritas Health Innovation, Melbourne, Australia) for screening.^21^ The study selection process was documented and presented in a PRISMA flow diagram.^20^ Data charting from the included sources of evidence was done using forms that have been tested by the team before their use and was done independently by two reviewers.

### Data Items

For each study, the data items extracted were study design, sample size, patient demographics (sex, median age), treatment characteristics (proportion of patients undergoing curative intent surgery, neoadjuvant radiotherapy and/or chemotherapy, exenterative surgery, or multivisceral resection), R0 resection rate, oncological outcomes (local or locoregional recurrence rate, overall survival, any other reported oncological outcomes), and biomarker data (biomarker/s evaluated, time point(s) at which biomarkers were evaluated, association with reported oncological outcomes).

### Quality Assessment

A formal quality assessment was not conducted because this scoping review was designed to describe the available literature rather than to evaluate study quality.

## Results

**Fig. 1:**
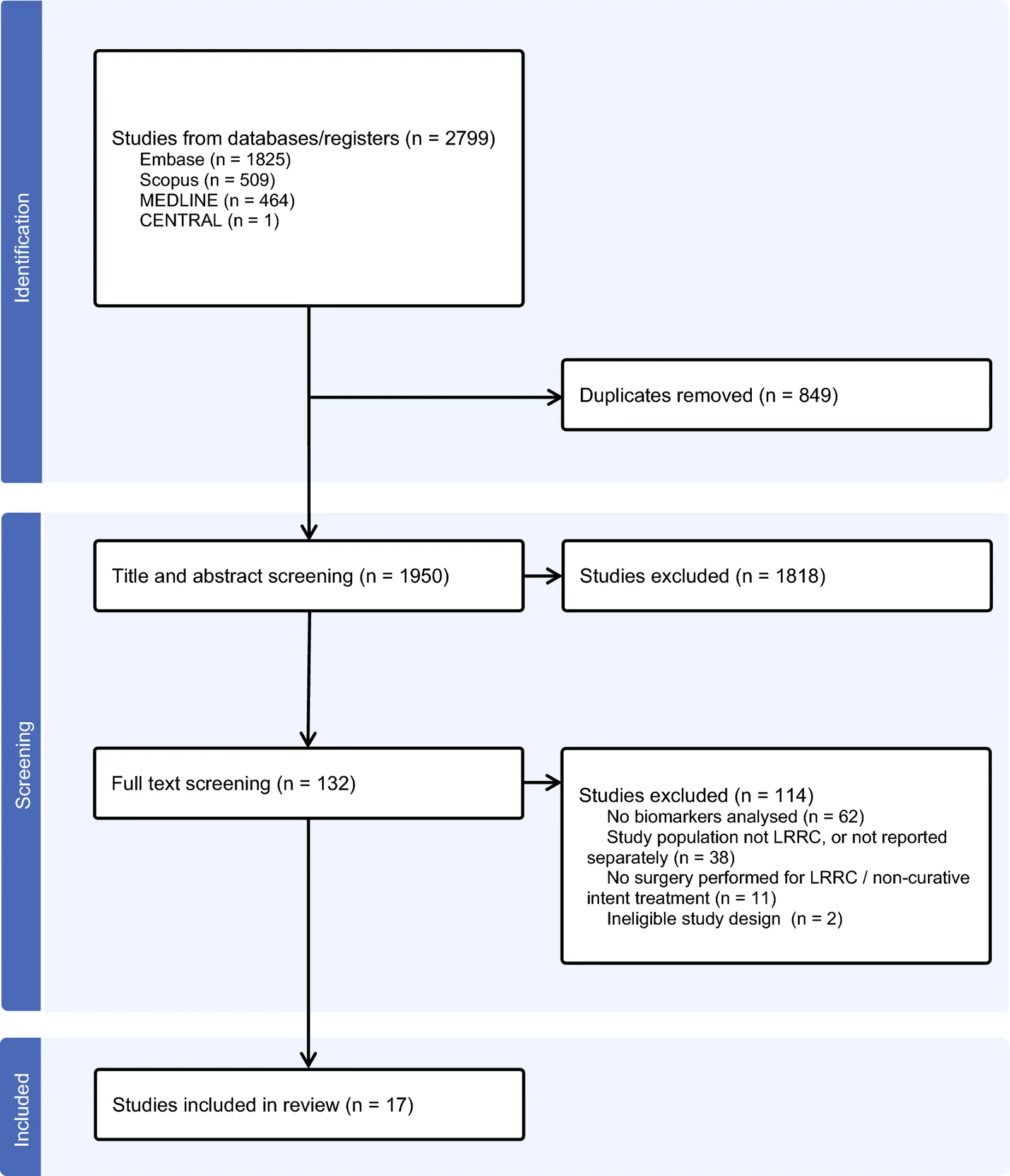
Preferred reporting items for systematic reviews and meta‐analyses (PRISMA) flow diagram of the study identification and screening process^20^

The search identified 2799 articles. After removal of duplicates, studies were screened against the inclusion and exclusion criteria, resulting in 17 articles being included in the final review (Fig. 1). Sixteen of 17 studies were retrospective cohort studies, and 1 was a protocol for an ongoing prospective cohort study.^22^ Included study characteristics are summarized in Table 1. Sample size ranged from 21 to 213 patients,^23,24^ and median patient age ranged from 50 to 65 years (range 19–91 years).^24,25^ There was considerable variation in the rate of neoadjuvant radiotherapy (10–100% of patients) and neoadjuvant chemotherapy (0.6–44%) between studies.^26,27^ Rate of exenteration or multivisceral resection ranged from 6% to 75%.^25,29^ The rate of curative-intent surgery ranged from 60% to 100%,^23,28,30–33^ and R0 resection was achieved in 48–100% of cases.^23,25^ Locoregional recurrence rates following curative-intent surgery for LRRC ranged from 15% to 52%,^27,32^ with the length of follow-up varying from 3 to 5 years. The 5-year overall survival varied from 22% to 52%.^24,29^ Fifteen studies (88%) evaluated serum carcinoembryonic antigen (CEA), two studies evaluated serum CEA doubling time (CEA-DT), two studies evaluated serum carbohydrate antigen 19-9 (CA19-9), and three studies evaluated other biomarkers (Table 1). Of the two studies that evaluated CA19-9, one included patients with distant disease (liver and paraaortic lymph node metastases but not peritoneal or lung disease).^24,31^

**Table 1:** Characteristics of included studies evaluating molecular biomarkers of oncologic outcome in patients undergoing curative-intent treatment for locally recurrent rectal cancer

First author and publication year	Country	Study design	Total LRRC sample size (number who underwent surgery)	Sex (male :female)	Age, median, years (range	Metastatic disease (%)	Curative intent surgery (%)	Neoadjuvant radiotherapy (%)	Neoadjuvant chemotherapy (%)	Exenteration/multivisceral resection (%)	R0 resection (%)	Local/locoregional recurrence (%)	Overall survival	Biomarker
Asoglu 2007^25^	Turkey	Retrospective cohort	50 (50)	33:17	50 (26–79)	0 (0)	36 (72)	NR	Not reported	3 (6)	24 (48)	8 (22)	Median 19 months (range 3–81)	Serum CEA
Bedrosian 2006^28^	USA	Retrospective cohort	85 (85)^)#^	49:36	58 (22–83)	0 (0)	85 (100)	23 (27)	37 (44)	NR	65 (76)	31 (36)	36% (5-year)	Serum CEA
														Tumour p53
														Tumour ki-67
														Tumour bcl-2
Cho* 2017^49^	Korea	Retrospective cohort	745 (177)	314:165	57 (19–86)	NR	NR	NR	NR	NR	NR	NR	40% (5-year, whole cohort)	Serum CEA
Hashimoto 2023^22^	Japan	Prospective cohort (protocol)	–	–	–	0 (0)	–	–	–	–	–	–	–	Plasma ctDNA
Henry 2007^36^	USA	Retrospective cohort	90 (90)	56:34	62 (33–91)	0 (0)	73 (81)	NR	NR	37 (41)	51 (57)	35 (48)	40% (5-year)	Serum CEA
Matsuyama* 2021^24^	Japan	Retrospective cohort	498 (213)	338:160	65 (235 <65, 263 ≥65)	0 (0)	213 (100)	NR	NR	NR	NR	NR	52% (5-year)	Serum CEA
														Serum CA19-9
Nagata 2011^37^	Japan	Retrospective cohort	27 (27)	NR	NR	NR	NR	NR	NR	NR	NR	NR	Median 24 months (range 3–71)	Tumour CD133 and CD44
Ogawa 2015^30^	Japan	Retrospective cohort	41 (41)^##^	33:8	NR. 20 ≥60, 21 ≤59).	0 (0)	41 (100)	25 (61)	NR	21 (51)	NR	16 (39)	NR	Serum CEA
Pacelli 2010^33^	Italy	Retrospective cohort	58 (58)	32:26	NR	0 (0)	35 (60)	46 (79)	NR	5 (9)	25 (43)	9 (26)	40% (5-year)	Serum CEA
Paku 2023^31^	Japan	Retrospective cohort	90 (90)	56:34	59 (IQR 53–66)	4 (4)	90 (100)	46 (51)	9 (10)	45 (50)	NR	44% (5-year)	NR	Serum CEA
														Serum CA19-9
Sakamoto 2022^32^	Japan	Retrospective cohort	33 (33)	23:10	60 (43–82)	0 (0)	33 (100)	7 (21)	NR	NR	33 (100)	5 (15)	63% (3- year)	Serum CEA-DT
Tanaka 2008^35^	Japan	Retrospective cohort	43 (43)	23:20	59 (32–80)	8 (19)	27 (63)	NR	NR	17 (40)	27 (63)	8 (30)	51% (5-year)	Serum CEA-DT
Tanaka 2017^26^	Japan	Retrospective cohort	180 (180)	121:59	56 (25–79)	24 (13)	171 (95)	18 (10)	1 (0.6)	127 (71)	134 (74)	29% (3-year)	39% (5-year)	Serum CEA
Uemura 2011^23^	Japan	Retrospective cohort	21 (21)	15:6	59 (47–77)	13 (62)	21 (100)	0 (0)	0 (0)	13 (62)	21 (100)	11 (52).	NR	Serum CEA
Wiig 2008^27^	Norway	Retrospective cohort	150 (150)	92:58	NR	0 (0)	124 (83)	150 (100)	NR	53 (35)	66 (44)	52% (5-year)	27% (5-year)	Serum CEA
Zhang 2020^29^	China	Retrospective cohort	111 (111)	59:52	58 (30–82)	0 (0)	103 (93)	45 (41)	NR	83 (75)	83 (75)	42 (41)	22% (5-year)	Serum CEA
Zhao* 2012^34^	China	Retrospective cohort	90 (41)	54:36	59 (27–76)	0 (0)	NR	10 (24)	NR	9 (22)	36 (88)	NR	31% (5-year, whole cohort)	Serum CEA

Data presented as frequency (percentage) unless otherwise specified

NR, not reported; CEA, carcinoembryonic antigen; CEA-DT, CEA doubling time; CA19-9, carbohydrate antigen 19-9

*Studies in which the cohort for the biomarker analysis included both patients who underwent salvage surgery and those who did not

#Samples from only 26 of 85 patients undergoing curative resection were available for immunohistochemical analysis with p53, ki-67, and bcl-2

##All were posterior invasive LRRC

### CEA

Elevated serum CEA was most commonly defined as ≥ 5 ng/ml (in six studies) and ≥ 10 ng/ml (in three studies). The timepoint of CEA measurement was at the time of diagnosis of, or prior to, surgery for LRRC in all included studies. Eleven studies assessed the association between CEA and overall survival (OS), of which ten studies reported that an elevated serum CEA was a negative predictor of OS (Table 2). One study reported no association between serum CEA and OS, however this multivariate analysis also included patients who did not undergo salvage surgery for LRRC.^34^ Short CEA-DT was reported to be a negative independent predictor of OS in one study (CEA-DT < 150 days; OR 0.268, 95% CI 0.084–0.861, *p* = 0.0270),^35^ and of recurrence-free survival (RFS) in another (CEA ≤ 210 days; HR 0.32, 95% CI 0.12–0.87; *p* = 0.026).^32^

**Table 2:** Findings of studies that evaluated serum CEA as a biomarker of oncologic outcome in patients undergoing surgery for locally recurrent rectal cancer

First author and publication year	Biomarker measurement	Time point	Association with OS	Other evaluated outcomes
Asoglu 2007^25^	CEA > 4 (units not provided)	Diagnosis of recurrence	Negative predictor (*p* = 0.004)	Curative resection: negative predictor (*p* = 0.02)
Bedrosian 2006^28^	CEA > 1.5 (units not provided)	Preoperative	Negative independent predictor (HR 2.41, 95% CI 1.19–4.89, *p* = 0.02)	DSS: no association (*p* = 0.08)
Cho* 2017^49^	CEA ≥ 5 ng/mL	Diagnosis of recurrence	Negative independent predictor (HR 2.508, 95% CI 2.014–3.124, *p* < 0.001)	Salvage treatment: negative predictor (HR 2.709, 95% CI 2.119–3.463, *p* < 0.001)
Henry 2007^36^	CEA > 20 ng/dL	Diagnosis of recurrence	Negative independent predictor (median 16 versus 63 months, *p* = 0.0001 and survival >18 months (36% versus 83%, *p* = 0.03)	Local control rate: no association with (*p* = 0.08)
Matsuyama* 2021^24^	CEA ≥ 5 ng/mL	Diagnosis of recurrence	Negative independent predictor (HR = 1.35, 95% CI 1.02–1.77, *p* = 0.03)	DSS: no association (*p* = 0.055)
Ogawa 2015^30^	CEA > 10 ng/mL	Preoperative		LRFS: no association (*p* = 0.24)
Pacelli 2010^33^	CEA ≥ 5 ng/mL	Preoperative	Negative independent predictor (RR 1.16, 95% CI 0.22–6.26, *p* < 0.001)	
Paku 2023^31^	CEA ≥ 5 ng/mL	Preoperative		RFS: no association (*p* = 0.98)
				CSS: no association (*p* = 0.09)
				Postoperative CEA was associated with shorter RFS (HR 2.18; 95% CI, 1.06–4.45; *p* = 0.03) and CSS (HR 2.32; 95% CI, 1.00–5.37; *p* = 0.04)
Sakamoto 2022^32^	CEA-DT ≤ 210 days	Diagnosis of recurrence		RFS: negative independent predictor (HR 0.32, 95% CI 0.12–0.87, *p* = 0.026)
Tanaka 2008^35^	CEA-DT < 150 days	Preoperative	Negative independent predictor (OR 0.268, 95% CI 0.084–0.861, *p* = 0.0270)	
Tanaka 2017^26^	CEA > 10 ng/ml	Diagnosis of recurrence	Negative independent predictor (HR 1.86, 95% CI 1.21–2.84, *p* = 0.004	Relapse-free survival: negative independent predictor (HR 1.82, 95% CI 1.29–2.58, *p* = 0.01)
Uemura 2011^23^	CEA > 10 ng/ml	Preoperative		LRFS: no association (*p* = 0.3362)
Wiig 2008^27^	CEA > 5 ng/ml	Diagnosis of recurrence	Negative independent predictor (*p* = 0.016)	R0 resection rate: negative independent predictor (HR 0.231, 95% CI 0.115–0.465, *p* = 0.010)
				Local recurrence: negative independent predictor (*p* = 0.04).
Zhang 2020^29^	CEA ≥ 15 µg/L	Preoperative	Negative independent predictor (HR 1.857, 95% CI 1.157–2.980, *p* = 0.010)	
Zhao* 2012^34^	CEA > 5 ng/ml	Diagnosis of recurrence	No association (*p* = 0.853), however analysis included whole cohort in which 41 (46%) patients underwent surgery	

*Studies in which the cohort for the biomarker analysis included both patients who underwent salvage surgery and those who did not

CEA, serum carcinoembryonic antigen; CEA-DT, serum CEA-doubling time; OS, overall survival; DSS, disease-specific survival; LRFS, local recurrence-free survival; RFS, recurrence-free survival; CSS, cancer-specific surviva; HR, hazard ratio (HR > 1.00: higher likelihood of event compared with reference); OR, odds ratio (OR > 1.00: higher likelihood of event compared with reference); RR, risk ratio (RR > 1.00: higher likelihood of event compared to reference); CI, confidence interval (95% CI includes 1.00; result not statistically significant, 95% CI does not include 1.00; result statistically significant)

Elevated CEA > 10 ng/ml,^26^ but not ≥ 5 ng/ml,^31^ was reported to be a negative independent predictor of relapse-free survival (HR 1.82, 95% CI 1.29–2.58, *p* = 0.01). Wiig and colleagues reported that elevated CEA was a negative independent predictor of local recurrence (*p* = 0.04).^27^ However, elevated CEA has not been significantly associated with disease-specific survival (DSS)^24,28^ or local recurrence-free survival (LRFS).^23,30,36^ Two studies reported that elevated CEA was a negative predictor for curative resection.^25,27^

### Other Serum Biomarkers

Elevated serum CA19-9 at the time of diagnosis of LRRC was associated with a shorter OS (HR = 1.58, 95% CI 1.17–2.13, *p* = 0.003), DSS (*p* = 0.002), and cancer-specific survival (CSS; *p* = 0.03) (Table 3).^24,31^ The ongoing prospective study by Hashimoto and colleagues is the first study to assess the association between circulating tumour DNA (ctDNA) after surgery for LRRC and minimal residual disease (MRD), with results pending at the time of this review.^22^

**Table 3:** Findings of studies which evaluated other potential biomarkers of oncologic outcome in patients undergoing surgery for locally recurrent rectal cancer

First author and publication year	Biomarker quantification	Time point/s	Association with OS	Other evaluated outcomes
Bedrosian 2006^28^	Tumour tissue p53 (>10% versus <10%)	Post-surgery	No association (*p* = 0.23, *p* = 0.93, and *p* = 0.94, respectively).	DSS: no association (*p* = 0.17, *p* = 0.96, and *p* = 0.87, respectively)
	Tumour tissue ki-67 (>50% versus 10–50% versus <10%)			
	Tumour tissue bcl-2 (>10% versus <10%)).			
Hashimoto 2023^22^	ctDNA-positive	Pre- and post-	Study underway	Study underway
		Neoadjuvant treatment	Evaluating OS	Evaluating RFS, LRFS, R0 resection rate, and pCR
		Pre- and post-surgery		
		Post-adjuvant chemotherapy		
Matsuyama* 2021^24^	CA19-9 ≥ 37 ng/ml	Diagnosis of recurrence	Negative independent predictor (HR=1.58, 95% CI 1.17–2.13, *p* = 0.003).	DSS: negative independent predictor (HR=1.65, *p* = 0.002).
Nagata 2011^37^	Tumour tissue CD133 and CD44 positive (immunohistochemistry)	Post-surgery	Presence of both CD133 and CD44: negative predictor of OS (0% versus 25% (5-year), *p* = 0.0225)	NR
			CD133 alone: no association (*p* = 0.5748)	
			CD44 alone: no association (*p* = *p* = 0.088)	
Paku 2023^31^	CA19-9 ≥ 37 U/ml	Pre- and post-surgery	NR	CSS: negative predictor (HR 2.42; 95% CI, 1.08–5.40, *p* = 0.03)
				RFS: no association (*p* = 0.09)
				Postoperative CA19-9 not associated with RFS (*p* = 0.751) or CSS (*p* = 0.865)

*Studies in which the cohort for the biomarker analysis included both patients who underwent salvage surgery and those who did not

CA19-9, serum carbohydrate antigen 19-9; OS, overall survival; DSS, disease-specific survival; LRFS, local recurrence-free survival; RFS, recurrence-free survival; CSS, cancer-specific survival; pCR, pathology complete response rate; HR, hazard ratio (HR > 1.00: higher likelihood of event compared with reference); OR, odds ratio (OR > 1.00: higher likelihood of event compared with reference); RR, risk ratio (RR > 1.00: higher likelihood of event compared with reference); CI, confidence interval (95% CI includes 1.00; result not statistically significant, 95% CI does not include 1.00; result statistically significant)

### Tumour Tissue-Based Biomarkers

Two studies that assessed tumour tissue-based markers obtained intraoperatively were identified. Nagata and colleagues reported that the presence of both CD133 and CD144 on tumour tissue immunohistochemistry negatively predicted OS on Kaplan–Meier survival analysis, without including CEA as a confounder, and neither marker alone was significantly associated with OS.^37^ Bedrosian and colleagues reported no association between tumour tissue staining for p53, Ki-67, or Bcl-2 and either OS or DSS (Table 3).^28^

No studies evaluating stool, saliva, urine, peritoneal fluid, or other biomarkers were identified.

## Discussion

This scoping review demonstrates that the current evidence base for molecular biomarkers in patients undergoing curative intent surgical treatment for LRRC is limited. Among the 17 included studies, only a small number of conventional biomarkers were evaluated, most commonly CEA. Elevated serum CEA is associated with poorer overall and recurrence-free survival in patients undergoing surgery for LRRC. Serum CEA-DT, elevated serum CA19-9, and the presence of both CD133 and CD44 in tumour tissue have also been identified as poor prognostic markers. However, it is important to emphasize that these findings do not reflect analyses designed to evaluate biomarker performance. In almost all studies, biomarkers were included as covariates within multivariable models designed to identify clinical predictors of oncologic outcome, rather than being assessed as candidate prognostic markers with appropriate measures of predictive performance such as discrimination, calibration, or incremental prognostic value. Consequently, the clinical utility of the identified biomarkers for risk stratification is uncertain. Importantly, none of the molecular biomarkers used routinely in contemporary care of primary and metastatic colorectal cancer patients (such as KRAS, NRAS, BRAF, mismatch repair deficiency, microsatellite instability, or ctDNA) have been evaluated in the LRRC population, and no DNA or RNA sequencing data were identified. There is also little evidence of ongoing work in this area, with the exception of a prospective cohort study evaluating ctDNA by Hashimoto and colleagues.^22^ Collectively, these findings highlight not only the narrow scope of biomarkers studied to date but also the absence of dedicated biomarker-driven research in this field.

Despite major advances in surgical technique that have transformed the management of LRRC from predominantly palliative to offering the potential for long-term survival, distant recurrence remains the most common pattern of failure and the main limitation to durable oncologic control.^7^ Improvements in preoperative imaging, centralization to high-volume centers, and advances in surgical technique over recent decades have increased rates of R0 resection and improved perioperative outcomes. However, in an era where en bloc pelvic bone and neurovascular resection is now performed routinely at specialized centres, it is unlikely that even more radical “higher and wider” resections will translate to meaningful reductions in the risk of systemic relapse. There is also wide variation and ongoing uncertainty around the role of induction chemotherapy, re-irradiation, and intraoperative radiotherapy as part of multimodal treatment of LRRC. Despite routine use of induction chemotherapy in some centers, available data have not demonstrated improvements in R0 resection, nor disease-free or overall survival rates with this approach.^13,38^ Re-recurrence rates (local and distant) remain as high as 50–55% even after R0 resection.^1,4,7,8^ Without improved risk stratification to identify patients at greatest risk of disease recurrence, inform biologically driven (neo)adjuvant strategies, and measure therapeutic response to those neoadjuvant treatments, it is unlikely that long-term oncologic outcomes in LRRC will substantially improve beyond current benchmarks.^39,40^

Although prognostic and predictive biomarkers have been increasingly characterized in colorectal cancer more broadly (Table 4),^41–43^ LRRC likely represents a biologically and clinically distinct entity from primary rectal cancer, and findings for primary rectal cancer may not necessarily be applicable to LRRC.^16,17^ Whether, for example, the established association between RAS/RAF mutations and early treatment failure in advanced primary rectal cancer also applies to LRRC should be specifically evaluated rather than extrapolated.^44,45^ Patients with LRRC, by definition, have had prior treatment exposures, have altered patho-anatomy, and require beyond-total mesorectal excision (TME) surgery for complete excision. The different patterns of failure in LRRC and the fact that these cancers have failed prior therapy support the concept of a fundamentally different disease process compared with treatment-naive primary tumours.^7^ Consequently, conventional frameworks such as TNM staging are not applicable or validated in a LRRC population and therefore may offer limited prognostic discrimination. Two recent large, contemporary series have demonstrated the difficulty in reliably predicting long-term oncologic outcomes following surgery for LRRC.^46,47^ The findings of this scoping review are consistent with a previous review by Gould and colleagues that focused on traditional pathological predictors rather than molecular biomarkers, yet also identified no studies evaluating molecular determinants of outcomes in LRRC.^10^

**Table 4:** Established and emerging molecular biomarkers that have been evaluated in broader colorectal cancer populations (particularly the stage IV setting) which may serve as a list as candidate biomarkers for de novo studies in patients with LRRC^41–43^

Established biomarkers used in clinical care	Genomic: KRAS mutations, NRAS mutations, BRAF V600E, HER2 amplification, NTRK fusions, gene expression panels (e.g. Oncotype DX Colon Recurrence Score^®^)
	DNA repair/genomic instability: microsatellite unstable/deficient mismatch repair, tumour mutational burden
	Protein/serum: CEA, CA19-9
Validated but not universally adopted biomarkers	Epigenetic: MLH1 promoter methylation, SEPT9 methylation
	Transcriptomic: Consensus Molecular Subtypes
	Tumour microenvironment/immune: Immunoscore (CD3/CD8 infiltration), PD-L1 expression
Circulating/liquid biopsy biomarkers	Circulating tumour DNA
	Circulating tumour cells
	Cell-free DNA fragmentation patterns
	Exosomal RNA/proteins
Emerging molecular biomarkers	Non-coding RNA: microRNAs, long non-coding RNAs, circular RNAs
	Proteomic/metabolic: IGFBP2, DKK3, PKM2, VEGF, MMPs
	Microbiome-related: *Fusobacterium nucleatum*, gut microbiome signatures
Multi-omics/next-generation biomarkers	Integrated genomic–epigenomic signatures
	AI-derived histopathologic biomarkers
	Spatial transcriptomics
	Methylation panels (genome-wide CpG signatures)

These findings highlight a clear need to study LRRC as a distinct disease entity and to evaluate prognostic and predictive biomarkers specifically within this population. Potentially resectable LRRC remains an uncommon presentation, and this group of patients have historically been excluded from clinical trials as they fall outside the more conventional cohorts of either advanced primary rectal cancer or metastatic disease. LRRC case volumes are likely to be relatively low, even at high-volume units, and meaningful progress in biomarker discovery in the form of de novo biomarker studies will therefore require coordinated collaboration between centres. Dedicated translational research programs focused on LRRC are required, ideally utilizing established biomarker discovery roadmaps, with prospective biospecimen collection that is coordinated across centers that specialize in LRRC.^48^ This is the most realistic framework for systematic evaluation of molecular biomarkers and the development of biologically informed treatment strategies for patients with LRRC. Table 4 demonstrates the extensive biomarker work conducted in other colorectal cancer populations (particular the metastatic setting) and may serve as a list as candidate biomarkers for evaluation in de novo studies of patients with LRRC.^41–43^

The strengths of this review include its novel focus on molecular biomarkers specifically in the LRRC population and its comprehensive inclusion of all study designs from the past two decades, enabling identification of the scarce evidence on prognostic and predictive biomarkers in LRRC. Limitations of the scoping review design include limited assessment of study quality and heterogeneity across studies, particularly relating to patient population, neoadjuvant therapy use, extent of surgery, and R0 resection rates. This therefore limited comparability between studies and precluded formal meta-analysis. While this scoping review maps the existing evidence, the conclusions are hypothesis-generating and highlight the need for prospective, standardized, multicenter studies in this rare population.

## Conclusions

There is currently limited evidence evaluating molecular biomarkers of oncologic outcome in patients undergoing curative intent treatment for LRRC. Most available studies are retrospective and have demonstrated association between conventional markers such as serum CEA and CA19-9 and oncologic outcomes, but these have not been formally evaluated as prognostic biomarkers. The prognostic performance and clinical utility of these markers in LRRC therefore remains uncertain. Contemporary colorectal cancer biomarkers including tumour mutational profile and ctDNA are yet to be studied in this population, and no DNA or RNA sequencing data in LRRC are available. LRRC represents a biologically distinct, more challenging disease entity that carries a high risk of re-recurrence, which justifies de novo biomarker studies in this group. Biomarker-driven risk stratification may guide treatment selection and the development of personalized multimodal treatment strategies.

## Supplementary Information

Below is the link to the electronic supplementary material.

Supplementary file1 (DOCX 13 kb)

## References

[1] Steffens D, Solomon M, Young J, Koh C, Venchiarutti R, Lee P, et al. Cohort study of long-term survival and quality of life following pelvic exenteration. *BJS Open*. 2018;2(5):328–35. 10.1002/bjs5.75 PMC6156168 30263984

[2] Sutton PA, Brown KG, Ebrahimi N, Solomon MJ, Austin KK, Lee PJ. Long-term surgical complications following pelvic exenteration: operative management of the empty pelvis syndrome. *Colorectal Dis*. 2022;24(12):1491–7. 10.1111/codi.16238 35766998

[3] You YN, Skibber JM, Hu CY, Crane CH, Das P, Kopetz ES, et al. Impact of multimodal therapy in locally recurrent rectal cancer. *Br J Surg*. 2016;103(6):753–62. 10.1002/bjs.10079 PMC5407413 26933792

[4] Hagemans J, Van Rees J, Alberda W, Rothbarth J, Nuyttens J, van Meerten E, et al. Locally recurrent rectal cancer; long-term outcome of curative surgical and non-surgical treatment of 447 consecutive patients in a tertiary referral centre. *Eur J Surg Oncol*. 2020;46(3):448–54. 10.1016/j.ejso.2019.10.037 31761506

[5] Harris CA, Solomon MJ, Heriot AG, Sagar PM, Tekkis PP, Dixon L, et al. The outcomes and patterns of treatment failure after surgery for locally recurrent rectal cancer. *Ann Surg*. 2016;264(2):323–9. 10.1097/SLA.0000000000001524 26692078

[6] Koh CE, Solomon MJ, Brown KG, Austin K, Byrne CM, Lee P, et al. The evolution of pelvic exenteration practice at a single center: lessons learned from over 500 cases. *Dis Colon Rectum*. 2017;60(6):627–35. 10.1097/DCR.0000000000000825 28481857

[7] Vu JK, Brown KG, Solomon MJ, Ng K-S, Sutherland S, Mahon K, et al. Patterns and outcomes of disease recurrence after pelvic exenteration for locally recurrent rectal cancer. *Br J Surg*. 2025;112(8):znaf154. 10.1093/bjs/znaf154 40794624

[8] Wiig J, Dagenborg VJ, Larsen SG. Ten-year survival and pattern of recurrence in patients with locally recurrent rectal or sigmoid cancer undergoing resection. *Colorect Dis*. 2025;27(1):e17226. 10.1111/codi.17226 PMC11683314 39635974

[9] Collaborative P. Contemporary management of locally advanced and recurrent rectal cancer: views from the PelvEx collaborative. *Cancers*. 2022;14(1161):2022. 10.3390/cancers14051161 PMC8909015 35267469

[10] Gould LE, Pring ET, Drami I, Moorghen M, Naghibi M, Jenkins JT, et al. A systematic review of the pathological determinants of outcome following resection by pelvic exenteration of locally advanced and locally recurrent rectal cancer. *Int J Surg*. 2022;104:106738. 10.1016/j.ijsu.2022.106738 35781038

[11] Kazi M, Desouza A, Saklani A. What are the preoperative predictors of a futile pelvic exenteration in rectal cancers? *Eur J Srug Oncol*. 2023;49(3):633–40. 10.1016/j.ejso.2022.10.022 36357296

[12] Brown KG, Coker DJ, Solomon MJ, Koh CE, Mahon K, Steffens D. The paradox of hope in surgery for advanced gastrointestinal malignancy: how do we define futility? *Ann Surg*. 2025;281(5):730–1. 10.1097/SLA.0000000000006575 39440417

[13] Nordkamp S, van Rees JM, van den Berg K, Mens DM, Creemers DM, Peulen HM, et al. Locally recurrent rectal cancer: oncological outcomes of neoadjuvant chemoradiotherapy with or without induction chemotherapy. *Br J Surg*. 2023;110(12):1637–40. 10.1093/bjs/znad214 37406084

[14] Vu JK, Brown KGM, Solomon MJ, Ng K-S, Mahon K, Le BK, et al. Oligometastatic disease is not an absolute contraindication to pelvic exenteration in selected patients with locally recurrent rectal cancer. *Dis Colon Rectum*. 2025;68(4):408–16. 10.1097/DCR.0000000000003613 39727315

[15] Kiran NS, Yashaswini C, Maheshwari R, Bhattacharya S, Prajapati BG. Advances in precision medicine approaches for colorectal cancer: from molecular profiling to targeted therapies. *ACS Pharmacol Transl Sci*. 2024;7(4):967–90. 10.1021/acsptsci.4c00008 PMC11019743 38633600

[16] Ryan EJ, Creavin B, Sheahan K. Delivery of personalized care for locally advanced rectal cancer: incorporating pathological, molecular genetic, and immunological biomarkers into the multimodal paradigm. *Front Oncol*. 2020;10:1369. 10.3389/fonc.2020.01369 PMC7456909 32923389

[17] De Mattia E, Polesel J, Mezzalira S, Palazzari E, Pollesel S, Toffoli G, et al. Predictive and prognostic value of oncogene mutations and microsatellite instability in locally-advanced rectal cancer treated with neoadjuvant radiation-based therapy: a systematic review and meta-analysis. *Cancers*. 2023;15(5):1469. 10.3390/cancers15051469 PMC10001009 36900260

[18] Lorena B, Gian LDA, Cinzia A, Francesco DM, Gioacchino L, Fabiola F, Francesca N. Potential predictive biomarkers in locally advanced rectal cancer treated with preoperative chemo-radiotherapy. *Acta Bio Medica Atenei Parmensis*. 2018;89(Suppl 9):102. 10.23750/abm.v89i9-S.7881 PMC6502185 30561402

[19] Peters MD, Marnie C, Tricco AC, Pollock D, Munn Z, Alexander L, et al. Updated methodological guidance for the conduct of scoping reviews. *JBI Evid Synth*. 2020;18(10):2119–26. 10.11124/JBIES-20-00167 33038124

[20] Tricco AC, Lillie E, Zarin W, O’Brien KK, Colquhoun H, Levac D, et al. PRISMA extension for scoping reviews (PRISMA-ScR): checklist and explanation. *Ann Intern Med*. 2018;169(7):467–73. 10.7326/M18-0850 30178033

[21] Innovation VH. Covidence systematic review software Melbourne, Australia [Available from: www.covidence.org.

[22] Hashimoto T, Tsukada Y, Ito M, Kanato K, Mizusawa J, Fukuda H, et al. Utility of circulating tumour DNA for prognosis and prediction of therapeutic effect in locally recurrent rectal cancer: study protocol for a multi-institutional, prospective observational study (JCOG1801A1, CAP-LR study). *BMJ Open*. 2023;13(8):e073217. 10.1136/bmjopen-2023-073217 PMC10432635 37586869

[23] Uemura M, Ikeda M, Yamamoto H, Kitani K, Tokuoka M, Matsuda K, et al. Clinicopathological assessment of locally recurrent rectal cancer and relation to local re-recurrence. *Ann Surg Oncol*. 2011;18(4):1015–22. 10.1245/s10434-010-1435-z 21082358

[24] Matsuyama T, Yamauchi S, Masuda T, Kikuchi A, Tokunaga M, Sugihara K, et al. Treatment and subsequent prognosis in locally recurrent rectal cancer: a multicenter retrospective study of 498 patients. *Int J Colorectal Dis*. 2021;36(6):1243–50. 10.1007/s00384-021-03856-3 33515308

[25] Asoglu O, Karanlik H, Muslumanoglu M, Igci A, Emek E, Ozmen V, et al. Prognostic and predictive factors after surgical treatment for locally recurrent rectal cancer: a single institute experience. *Eur J Surg Oncol*. 2007;33(10):1199–206. 10.1016/j.ejso.2007.02.026 17400423

[26] Tanaka M, Kanemitsu Y, Shida D, Ochiai H, Tsukamoto S, Nagino M, et al. Prognostic impact of intra-abdominal/pelvic inflammation after radical surgery for locally recurrent rectal cancer. *Dis Colon Rectum*. 2017;60(8):827–36. 10.1097/DCR.0000000000000853 28682968

[27] Wiig JN, Larsen SG, Dueland S, Giercksky KE. Preoperative irradiation and surgery for local recurrence of rectal and rectosigmoid cancer: prognostic factors with regard to survival and further local recurrence. *Colorectal Dis*. 2008;10(1):48–57. 10.1111/j.1463-1318.2007.01398.x 18028472

[28] Bedrosian I, Giacco G, Pederson L, Rodriguez-Bigas MA, Feig B, Hunt KK, et al. Outcome after curative resection for locally recurrent rectal cancer. *Dis Colon Rectum*. 2006;49(2):175–82. 10.1007/s10350-005-0276-5 16392024

[29] Zhang JL, Wu T, Chen GW, Wang PY, Jiang Y, Tang JQ, et al. Analysis on risk factors of the degree of radical resection and prognosis of patients with locally recurrent rectal cancer. *Zhonghua Wei Chang Wai Ke Za Zhi*. 2020;23(5):472–9. 10.3760/cma.j.cn.441530-20200207-00042 32842427

[30] Ogawa H, Uemura M, Nishimura J, Hata T, Ikenaga M, Takemasa I, et al. Preoperative chemoradiation followed by extensive pelvic surgery improved the outcome of posterior invasive locally recurrent rectal cancer without deteriorating surgical morbidities: a retrospective, single-institution analysis. *Ann Surg Oncol*. 2015;22(13):4325–34. 10.1245/s10434-015-4452-0 25893408

[31] Paku M, Uemura M, Kitakaze M, Miyoshi N, Takahashi H, Mizushima T, et al. Clinical significance of preoperative and postoperative serum CEA and carbohydrate antigen 19–9 levels in patients undergoing curative resection of locally recurrent rectal cancer. *Dis Colon Rectum*. 2023;66(3):392–400. 10.1097/DCR.0000000000002655 36649161

[32] Sakamoto J, Ozawa H, Nakanishi H, Fujita S. Usefulness of carcinoembryonic antigen doubling time in prognosis prediction after curative resection of locally recurrent rectal cancer: a retrospective study. *Dig Surg*. 2022;39(1):17–23. 10.1159/000520694 34749370

[33] Pacelli F, Tortorelli AP, Rosa F, Bossola M, Sanchez AM, Papa V, et al. Locally recurrent rectal cancer: prognostic factors and long-term outcomes of multimodal therapy. *Ann Surg Oncol*. 2010;17(1):152–62. 10.1245/s10434-009-0737-5 19834766

[34] Zhao J, Du CZ, Sun YS, Gu J. Patterns and prognosis of locally recurrent rectal cancer following multidisciplinary treatment. *World J Gastroenterol*. 2012;18(47):7015–20. 10.3748/wjg.v18.i47.7015 PMC3531688 23323002

[35] Tanaka K, Noura S, Ohue M, Seki Y, Yamada T, Miyashiro I, et al. Doubling time of carcinoembryonic antigen is a significant prognostic factor after the surgical resection of locally recurrent rectal cancer. *Dig Surg*. 2008;25(4):319–24. 10.1159/000158597 18818499

[36] Henry LR, Sigurdson E, Ross EA, Lee JS, Watson JC, Cheng JD, et al. Resection of isolated pelvic recurrences after colorectal surgery: long-term results and predictors of improved clinical outcome. *Ann Surg Oncol*. 2007;14(7):2000–9. 10.1245/s10434-006-9343-y 17431726

[37] Nagata T, Sakakura C, Komiyama S, Miyashita A, Nishio M, Murayama Y, et al. Expression of cancer stem cell markers CD133 and CD44 in locoregional recurrence of rectal cancer. *Anticancer Res*. 2011;31(2):495–500. 21378329

[38] Voogt E, Nordkamp S, Nieuwenhuijzen G, Creemers G, Peulen H, Rutten H, et al. Curative treatment of locally recurrent rectal cancer: is induction chemotherapy warranted? *Br J Surg*. 2021;108(6):e213–4. 10.1093/bjs/znab065 33709100

[39] Contemporary results from the PelvEx collaborative. improvements in surgical outcomes for locally advanced and recurrent rectal cancer. *Colorectal Dis*. 2024;26(5):926–31. 10.1111/codi.16948 38566456

[40] Brown KG, Solomon MJ, Koh CE, Sutton PA, Aguiar S Jr, Bezerra TS, et al. Defining benchmarks for pelvic exenteration surgery: a multicentre analysis of patients with locally advanced and recurrent rectal cancers. *Ann Surg*. 2025;282(6):1118–26. 10.1097/SLA.0000000000006348 38747145

[41] Agüera-Sánchez A, Peña-Ros E, Martínez-Martínez I, García-Molina F. Comprehensive landscape of diagnostic, prognostic and predictive biomarkers in colorectal cancer: from genomics to multi-omics integration in precision medicine. *J Personal Med*. 2026;16(1):48. 10.3390/jpm16010048 PMC12842678 41590540

[42] Ashouri K, Wong A, Mittal P, Torres-Gonzalez L, Lo JH, Soni S, et al. Exploring predictive and prognostic biomarkers in colorectal cancer: a comprehensive review. *Cancers*. 2024;16(16):2796. 10.3390/cancers16162796 PMC11353018 39199569

[43] Mannucci A, Goel A. Stool and blood biomarkers for colorectal cancer management: an update on screening and disease monitoring. *Mol Cancer*. 2024;23(1):259. 10.1186/s12943-024-02174-w PMC11575410 39558327

[44] Creavin B, Kelly ME, Noori J, Turner G, Guerra G, Behrenbruch C, et al. Early failure following pelvic exenteration: Who are the bad actors? *Surgeon*. 2025;23(4):211–5. 10.1016/j.surge.2025.02.010 40087056

[45] Bedrikovetski S, Traeger L, Fitzsimmons T, Price TJ, Ruszkiewicz AR, Vather R, et al. Association between RAS/BRAF mutations and complete response following total neoadjuvant therapy in patients with rectal cancer: a prospective multicentered study. *Ann Surg Oncol*. 2024;31(3):1681–9. 10.1245/s10434-023-14722-7 38071720

[46] Brown KGM, Ng KS, Solomon MJ, Vu J, Lo S, Sutton PA, et al. Preoperative predictors of priority outcomes of pelvic exenteration for locally recurrent rectal cancer: Results of the mixed-methods EviSurg study. *Eur J Surg Oncol*. 2026;52(1):111183. 10.1016/j.ejso.2025.111183 41240801

[47] Gould LE, Pring ET, Wallace A, Hodges N, Burns EM, Steele CW, et al. Clinical determinants of outcome following pelvic exenteration for locally advanced or locally recurrent rectal cancer. *Eur J Surg Oncol*. 2025;51(10):110347. 10.1016/j.ejso.2025.110347 40752227

[48] Lioumi M, Newell D. CR-UK biomarker roadmaps. *Clin Cancer Res*. 2010;16(19_Supplement):B33–B33.

[49] Cho WK, Choi DH, Park HC, Park W, Yu JI, Park YS, et al. Elevated CEA is associated with worse survival in recurrent rectal cancer. *Oncotarget*. 2017;8(62):105936–41. 10.18632/oncotarget.22511 PMC5739691 29285304

