# Supplementary material for: Locally Recurrent Rectal Cancer in the Molecular Era: A Scoping Review of Biomarkers of Oncologic Outcome following Salvage Surgery
Source: Ann Surg Oncol. 2026 Jul 28;33(12):10629–40. doi: 10.1245/s10434-026-20116-2 (PMC13675342; doi:10.1245/s10434-026-20116-2)
Supplement: Supplementary file 1 [file 10434_2026_20116_MOESM1_ESM.docx]

**SUPPLEMENTARY INFORMATION**

**MEDLINE search strategy**

1. Exp Biomarkers, Tumor/ or molecular diagnostic techniques/ or exp immunohistochemistry/ or proteomics/ or proteogenomics/ or genomics/ or epigenomics/ or metabolomics/ or lipidomics or multiomics/ or exp transcription, Genetic/ or tumor microenvironment/ or exp mutation/
2. (“molecular biomarker” or biomarker or “T cell” or macrophage or “B cell” or “natural killer cell” or “dendritic cell” or “antigen presenting cell” or “cancer associated fibroblast” or immunohistochemistry or epigenomic* or lipidomic* or multiomic* or metabolomic* or proteomic* or gene* or genomic* or transcript* or mutat*).mp
3. 1 or 2
4. Colorectal Surgery/ or exp Proctectomy/ or Pelvic Exenteration/
5. (Surger* or resect* or excis* or exent* or radical* or operat* or procedur*).mp
6. 4 or 5
7. Rectal neoplasms/
8. (rect* adj3 (cancer* or neoplas* or malig* or tumo?r*)).mp
9. 7 or 8
10. (recur* adj8 rect*).mp
11. 3 and 6 and 9 and 10
12. Limit 11 to yr= “2005-Current”

460 results

**EMBASE search strategy**

1. exp tumor marker/ or exp immunohistochemistry/ or exp epigenetics/ or epigenome/ or genome/ or immunomics/ or lipidomics/ or metabolomics/ or multiomics/ or peptidomics/ or proteomics/ or transcriptomics/ or molecular genetics/ or immunogenomics/ or oncogenomics/ or genomics/ or exp tumor microenvironment/ or exp mutation/
2. (“molecular biomarker” or biomarker or “T cell” or macrophage or “B cell” or “natural killer cell” or “dendritic cell” or “antigen presenting cell” or “cancer associated fibroblast” or immunohistochemistry or epigenomic* or lipidomic* or multiomic* or metabolomic* or proteomic* or gene* or genomic* or transcript* or mutat*).mp
3. 1 or 2
4. Exp rectum surgery/ or Proctectomy/ or Pelvic Exenteration/
5. (Surger* or resect* or excis* or exent* or radical* or operat* or procedur*).mp
6. 4 or 5
7. (Exp rectum cancer/ or exp rectum carcinoma/ or exp rectum tumor/)
8. (rect* adj3 (cancer* or neoplas* or malig* or tumo?r*)).mp
9. 7 or 8
10. (recur* adj8 rect*).mp
11. 3 and 6 and 9 and 10
12. Limit 11 to yr= “2005-Current”

1,765 results

**COCHRANE CENTRAL REGISTER OF CONTROLLED TRIALS search strategy**

1. Exp Biomarkers, Tumor/ or exp T-Lymphocytes/ or exp Killer Cells, Natural/ or exp Macrophages/ or exp B-Lymphocytes/ or exp Antigen-Presenting Cells/ or exp Cancer-Associated Fibroblasts/ or exp Lipidomics/ or exp Precision Medicine/ or exp Transcription, Genetic/ or exp Gene expression/ or exp Cellular microenvironment/ or exp Tumor Microenvironment/ or exp Mutation/ or exp Immunohistochemistry/
2. (“molecular biomarker” or biomarker or “T cell” or macrophage or “B cell” or “natural killer cell” or “dendritic cell” or “antigen presenting cell” or “cancer associated fibroblast” or immunohistochemistry or epigenomic* or lipidomic* or multiomic* or metabolomic* or proteomic* or gene* or genomic* or transcript* or mutat*)
3. 1 or 2
4. Colorectal Surgery/
5. (Surger* or resect* or excis* or exent* or radical* or operat* or procedur*)
6. 4 or 5
7. Exp rectal neoplasms/
8. (rect* adj3 (cancer* or neoplas* or malig* or tumo?r*))
9. 7 or 8
10. (recur* adj8 rect*)
11. 3 and 6 and 9 and 10
12. Limit publication date from Jan 2005 to Oct 2025

1 result

**SCOPUS search strategy**

TITLE-ABS-KEY (“molecular biomarker” or molec* or biomarker or “T cell” or macrophage or “B cell” or “natural killer cell” or “dendritic cell” or “antigen presenting cell” or “cancer associated fibroblast” or immunohistochemistry or epigenomic* or lipidomic* or multiomic* or metabolomic* or proteomic* or gene* or genomic* or transcript* or mutat*)

AND

TITLE-ABS-KEY (Surger* or resect* or excis* or exent* or radical* or operat* or procedur*)

AND

TITLE-ABS-KEY (rect* W/3 (cancer* or neoplas* or malig* or tumo?r*))

AND

TITLE-ABS-KEY (recur* W/8 rect*)

Filter date 2005 – 2025

504 results
